# Changes observed in prostate biopsy practices in an inner city hospital with a high risk patient population following the 2012 USPSTF PSA screening recommendations

**DOI:** 10.1590/S1677-5538.IBJU.2017.0348

**Published:** 2018

**Authors:** Andrew W. Tam, Johnathan A. Khusid, Igor Inoyatov, Adan Z. Becerra, Jonathan Davila, Jyoti D. Chouhan, Jeffrey P. Weiss, Llewellyn M. Hyacinthe, Brian K. McNeil, Andrew G. Winer

**Affiliations:** 1 Department of Urology, University Hospital of Brooklyn, State University of New York Downstate College of Medicine, Brooklyn, NY, USA;; 2 Department of Public Health Sciences and Division of Epidemiology, University of Rochester Medical Center, Rochester, NY, USA;; 3 Department of Surgery, University of Rochester Medical Center, Surgical Health Outcomes and Research Enterprise (SHORE), Rochester, NY, USA

**Keywords:** Mass Screening, Prostatic Neoplasms, Prostate-Specific Antigen

## Abstract

**Introduction:**

We compared characteristics of patients undergoing prostate biopsy in a high-risk inner city population before and after the 2012 USPSTF recommendation against PSA based prostate cancer screening to determine its effect on prostate biopsy practices.

**Materials and Methods:**

This was a retrospective study including patients who received biopsies after an abnormal PSA measurement from October 2008-December 2015. Patients with previously diagnosed prostate cancer were excluded. Chi-square tests of independence, two sample t-tests, Mann-Whitney U tests, and Fisher's exact tests were performed.

**Results:**

There were 202 and 208 patients in the pre-USPSTF and post-USPSTF recommendation cohorts, respectively. The post-USPSTF cohort had higher median PSA (7.8 versus 7.1ng/mL, p=0.05), greater proportion of patients who were black (96.6% versus 90.5%, p=0.01), and greater percentage of biopsy cores positive for disease (58% versus 29.5%, p<0.001). Multivariable analysis supported that the increase in PSA was independent of the increase in the proportion of patients who were black. The proportion of patients who were classified as D’Amico intermediate and high-risk disease increased in the post-USPSTF cohort and approached statistical significance (70.1% versus 58.8%, p=0.12).

**Conclusions:**

Our study suggests that the USPSTF recommendations may have led to an increase in pre-biopsy PSA as well as greater volume of disease. Also, a greater proportion of patients were being classified with intermediate or high risk disease. While the clinical significance of these findings is unknown, what the data suggests is somewhat *troubling.* Future research should further examine these changes in a larger cohort as well as resultant long-term outcomes.

## INTRODUCTION

In May 2012, the United States Preventative Services Task Force (USPSTF) issued a recommendation against prostate specific antigen (PSA)-based prostate cancer screening for men in the general United States population (1). This recommendation was rooted in two studies, the Prostate, Lung, Colorectal and Ovarian (PLCO) Cancer Screening Trial and the European Randomized Study of Screening for Prostate Cancer (ERSPC), neither of which reported a significant African American or Afro-Caribbean cohort (2, 3). The USPSTF acknowledges that the results of PLCO and ERSPC are difficult to generalize to the black population, and yields in suggesting that this high-risk population be evaluated under separate guidelines. Similarly, the National Comprehensive Cancer Network (NCCN) acknowledges that African Americans “have a higher incidence of prostate cancer, increased prostate cancer mortality, and earlier age of diagnosis compared to Caucasian-American men. However, the effects of earlier or more intensive screening on cancer outcomes and on screening-related harms in African-American men remain unclear” (4). It is uncertain how to best apply screening recommendations in a high-risk population such that the harms of overscreening are balanced with the benefits of early detection of clinically significant disease.

Given the increased risk for disease as well as predisposition for more aggressive disease in African Americans (4-8), we hypothesize that prostate biopsy practices in an inner-city hospital serving a predominately black population were not changed in response to the USPSTF guidelines. Thus, we sought to characterize prostate biopsy patterns in a single academic institution where the patients mostly identify as Caribbean, Caribbean American, African, and African American.

## MATERIALS AND METHODS

After obtaining IRB approval (#793946-1) we performed a retrospective chart review of patients who received initial prostate biopsies at a single institution from October 27, 2008 to December 15, 2015. All patients received a 12-core systematic transrectal ultrasound guided biopsy. The primary population was divided into patients biopsied prior to May 22, 2012 and patients biopsied on or after that date. History of prostate cancer and absence of data on age, PSA, race, specialty of provider that initiated PSA screening, or results of prostate biopsy were excluded. We collected age, race, PSA prior to biopsy, provider specialty who initiated screening, and biopsy results (Gleason score, number of cores positive). PSA values were unadjusted for finasteride use. Digit rectal exam findings were unavailable for a significant portion of our population, and so were excluded from our analysis.

Chi-square tests of independence, two sample t-tests, and Mann-Whitney U tests were performed to compare patient demographic and clinical factors in both cohorts. Analysis included age, race, PSA level, whether a primary care physician (PCP) or urologist initiated PSA screening, and diagnosis of biopsy. Subsequently, a conditional multivariable logistic regression model was built to characterize the independent effect of each factor on undergoing a biopsy in the pre-recommendation period (group-1) versus the post-recommendation period (group-2). The model estimated odds ratios and 95% confidence intervals for each predictive factor. Standard regression assumptions were verified graphically. More specifically, plots were used to confirm that independent variables were linear with the log odds; variable transformations were not necessary. Regarding missing data, 1% of patients had a missing data point that was used for analysis. Imputation of missing data was achieved using multivariate imputation by chained equations (9).

Among patients who had a positive biopsy, the percent cores positive, Gleason score, and D’Amico Risk Group (low vs. intermediate vs. high) were compared between the two time periods using a chi-square test of independence, Mann-Whitney U test, and Fisher's exact test. All analyses were conducted using in R Statistical Software (Foundation for Statistical Computing, Vienna, Austria).

## RESULTS

Overall, 202 patients underwent prostate biopsy prior to the USPSTF recommendation (group-1) and 208 patients underwent prostate biopsy after the USPSTF recommendation (group-2). Of note, MRI and fusion biopsy are not used in the diagnosis of prostate cancer in this institution, and as so these are unlikely to serve as a confounder. In univariate analysis, there was no significant difference in the mean age, whether a PCP or urologist initiated PSA-based screening, or the percentage of positive biopsies between groups 1 and 2 (Table-1). Group-2 had a significantly greater proportion of patients who were of African-American descent (96.6% versus 90.5%, p=0.01) (Table-1). Yet, the absolute difference in the number of African Americans biopsied is small (n=199 versus n=181) and has limited clinical significance. Additionally, median PSA was significantly greater in group-2 compared to group-1 (7.8 nanograms/milliliter (ng/mL) vs. 7.1 ng/mL, p=0.05) (Table-1). These results remained statistically significant in multivariable analysis (Table-2). Patients biopsied in the post-USPSTF period were 2.14 times more likely to be of African-American descent as compared to patients biopsied in the pre-USPSTF period (OR=2.14, 95% CI=1.05, 3.55). For every 1ng/mL increase in PSA in the post-USPSTF group, the odds of being biopsied increased 67% (OR=1.67, 95% CI=1.02, 2.38) relative to patients biopsied in the pre-USPSTF period.

**Table 1:** Characteristics of All Patients, Pre and Post USPSTF Guidelines.

Characteristic		Pre-USPSTF N= 202 (49.5%)	Post-USPSTF N= 208 (50.5%)	p-value
Age (mean±sd)		64.6±8.3	64.1±7.7	0.51
PSA Level (Median, IQR)*		7.1 (8.8)	7.8 (9.1)	0.05
**Race**				0.01
	Black	181 (90.5%)	199 (96.6%)	
	Other	21 (9.5%)	9 (3.4%)	
**Decision to Screen PSA**				
	PCP	82 (41.2%)	90 (45.0%)	0.51
	Urologist	117 (58.8%)	110 (55.0%)	
**Positive Biopsies**				
	No	95 (47.0%)	106 (51.5%)	0.43
	Yes	107 (53%)	100 (48.5%)	

**Table 2:** Multivariable Analysis.

Risk Factor		Adjusted Odds Ratio (95% CI)	p-value
**Race**			
	Other	1.00 (Reference)	
	Black	2.14 (1.05, 3.55)	0.02
**PSA Level**			
	Per 1 point increase	1.67 (1.02, 2.38)	0.03

In a subset analysis, characteristics were compared among patients who had a positive biopsy in the pre and post-USPSTF period (n=107 and n=100, respectively) (Table-3). Our data showed that the median percentage of positive cores in group-2 was significantly greater than in group-1 (58% versus 29.5%, p<0.001). Though the analysis is underpowered, the data suggests that patients in group-2 exhibited worse D’Amico risk classification relative to patients in group-1. In the pre-USPSTF cohort, 44.1% and 58.8% of patients had low-risk and clinically significant disease respectively. In comparison, the post-USPSTF cohort exhibited 29.9% and 70.1% of patients with low-risk and clinically significant disease, respectively (p=0.12).

**Table 3:** Characteristics among those with a Positive Biopsy, Pre and Post USPSTF Guidelines.

Characteristic		Pre-USPSTF N= 107 (51.7%)	Post-USPSTF N= 100 (48.3%)	p-value
**% Cores positive (Median %)**		29.5%	58.0%	<0.001
**Gleason Score**				0.42
	6	53 (49.5%)	43 (43%)	
	7 (3+4)	19 (17.8%)	24 (24%)	
	7 (4+3)	20 (18.7%)	13 (13%)	
	8	5 (4.7%)	7 (7.0%)	
	9	9 (8.4%)	13 (13%)	
	10	1 (0.9%)	0 (0%)	
**D’Amico Risk Group**				0.12
	Low	44 (41.1%)	29 (29.9%)	
Intermediate and high risk		63 (58.8%)	68 (70.1%)	

## DISCUSSION

The demographics of our study population lend insight on the effect of the USPSTF recommendation in a high-risk population. Previous literature has attempted to characterize the effect of the recommendation on screening and biopsy practices, but did so without a significant black population (10, 11). There is strong evidence to suggest that blacks are more likely to develop prostate cancer in their lifetime and that their disease tends to progress more quickly as well (6-8, 12, 13). Given these considerations, we sought to determine whether practice patterns have changed since May 2012.

In the study period following the publication of the recommendation, group-2 was significantly more likely to harbor a higher volume of disease, and was near-significantly more likely to harbor clinically significant D’Amico-classified intermediate or high-risk disease. Most strikingly, patients in group-2 with positive biopsies had almost double percentage of positive cores. This trend cannot be explained by the use of MRI or fusion guided biopsy as these technologies are not used at our institution. Similar results have been shown in the literature. Banerji et al. conducted a similar study, which evaluated 448 patients with prostate needle biopsies 30 months before and 310 patients with prostate needle biopsies 30 months after the USPSTF issued their recommendation. Their data showed that patients biopsied after the recommendations were more likely to have clinical T2b and T2c-T3a disease (adjusted p=0.012 and adjusted p=0.017, respectively). In addition, the post-USPSTF population was more likely to have D’Amico-classified high-risk prostate cancer (34% vs. 46%, adjusted p=0.027). Furthermore, the pre-USPSTF recommendation group was more likely to have a lower volume of disease: 22% of patients in the pre-recommendation group had less than 34% of biopsy cores showing cancer, whereas 29% of the post-recommendation group had less than 34% of biopsy cores showing cancer (unadjusted p=0.031) (10). Similar to our findings, their study suggests that since the USPSTF issued their recommendation, there has been an increase in the incidence of higher-risk and higher volume disease. Notably, the authors did not report on demographics and therefore it is difficult to confidently apply such findings to an innately high-risk population such as the one featured in our study.

There appears to be an interesting trend when considering the demographics of the two groups. Group-2 displayed an increase in the proportion of patients receiving a biopsy who were black, despite comparable age and number of patients receiving biopsy in the two cohorts. There was also a significant increase in the median PSA of biopsied individuals in the post-USPSTF recommendation group. The mechanism of this difference may be at the level of PSA screening, with provider focus on the high-risk population, or at the level of biopsy, with providers having a higher PSA threshold for biopsy. Another possibility is that unrecognized cultural influences, such as increased awareness of prostate cancer in the black community, has resulted in patients taking a more proactive role in screening for prostate cancer, with more patients asking to undergo PSA screening. Alternatively, these findings may be explained by shifts in the ethnic distribution of our hospital's catchment area over time. Of note, though the increase in proportion of black men amongst those undergoing prostate biopsy reached statistical significance, the increase in absolute number of black men undergoing prostate biopsy between the two groups was small (n=181 vs. n=199, pre-USPSTF recommendation group and post-USPSTF recommendation group respectively). Given the >90% black demographics in both groups, this significance and true mechanism of this finding remain unclear and require further research. In review, Perez et al. reported no significant differences between cohorts in the mean PSA and proportion of patients undergoing biopsy who were African American despite large differences in reported values (11). Perez et al. reported the mean PSA in the pre-USPSTF recommendation group as 7.7 ng/mL while the post-USPSTF recommendation group was 11ng/mL (p=0.31), and the proportion of patients who were African American in the pre-USPSTF recommendation group was 11.9 while in the post-USPSTF recommendation group was 18.8 (p=0.34). However, their study population was small, with 201 patients in the pre-USPSTF recommendation cohort and 212 in the post-USPSTF recommendation cohort. Nevertheless, the authors reported that on multivariable analysis, African Americans in the post-USPSTF cohort were nearly five times more likely to undergo biopsy when compared to African Americans in the pre-USPSTF cohort (OR 6.31, 95% CI 1.65-24.23; p=0.007) (11). The congruence of this data with ours is limited by the small African American cohort of their population. Regardless, there appears to be a focus on high-risk populations following the USPSTF recommendation.

Notably, there was no difference between the two groups in regards to the specialty of the provider who initiated PSA screening. As such, it is unlikely that recommendations from specialist groups such as the 2013 updated screening recommendations released by the American Urologic Association, would have had a major impact on screening practices. Prior to the USPSTF recommendation statement, slightly more patients received their initial PSA screening test from a urologist, which maintained true following the recommendation statement as well. This finding is somewhat expected, being that the USPSTF's recommendation was issued to the general public and not specialty focused. The stance on PSA-based screening has always mixed throughout the medical field, thus it is unexpected that urology practices differ greatly from non-urologists.

Our results must be considered through the scope of our limitations. We conducted a retrospective study, which is inevitably confounded by selection bias. In addition, our sample size may have been too small to lend significance to some of the trends revealed in our analysis. In particular, the difference in the proportion of patients in each D’Amico risk group trended towards significance. However, given that we collected data from only approximately 100 patients in each group, the presence or absence of a true difference remains unclear. Furthermore, our study assessed biopsy frequency on the basis of total biopsies performed. This metric is influenced by several factors including hospital resources. The rate of biopsy for patients with elevated PSA would strengthen out conclusions and should be a point of further study. Additionally, we were unable to sub-stratify patients by nationality as this data was unavailable in our chart review. Thus, we could not perform nationality or region-specific analyses on our diverse population, which is primarily composed of people from various countries in the Caribbean region and Africa. Of note, we selected the date of USPSTF guideline publication to stratify our two groups. This does not take into account a potential lag that may have occurred from the dissemination of the recommendations to their implementation. Despite these limitations, we feel that our study adds to the growing body of literature on the role of PSA screening and prostate biopsy in high-risk individuals, particularly black men.

We recommend further examination of practice patterns in order to determine whether the changes observed occurred due to provider selection of higher-risk patients for PSA screening or due to an increased PSA threshold to initiate biopsy. Additionally, we recommend research into outcome measures following the USPSTF recommendations to further understand the clinical implications of the USPSTF recommendations. We are aware that these recommendations are dynamic and that the USPSTF continues to work alongside the American Urologic Association and American Cancer Society to revise their proposals. In the most recent 2017 update, the USPSTF has recently relabeled PSA testing in men ages 55-69 years a grade “C” or recommended to be offered selectively to patients (14). Specifically, they recommend that the decision to receive PSA-based screening should be both individualized and shared between the clinician and patient. The risks and benefits of testing should be well understood by the patient before undergoing screening. Presently, USPSTF continues to recommend against PSA testing in males 70 years or older. With successful characterization of the trends in prostate biopsy practice patterns, we hope to guide practice patterns in the high-risk population with hopes of reducing overtreatment of clinically insignificant disease while continuing to aggressively treat clinically significant disease.

## CONCLUSIONS

In summary, we found that in the time since the USPSTF issued their recommendation against PSA-based prostate cancer screening, there has been a significant increase in the PSA of patients who have undergone prostate biopsy in our study population. Additionally, these patients were more likely to be black in comparison to the pre-recommendation cohort. Lastly, patients with a positive biopsy had higher volume of disease and were near-significantly more likely to have D’Amico-classified intermediate and high risk disease. More research is needed to identify the mechanisms underlying these observations. We also suggest further research into long-term outcomes in black men who are not undergoing PSA-based prostate cancer screening as per the USPSTF recommendations.

## ABBREVIATIONS

USPSTF: = United States Preventative Services Task Force
PSA: = Prostate specific antigen
NCCN: = National Comprehensive Cancer Network
PLCO (Cancer Screening Trial): = Prostate, Lung, Colorectal, Ovarian
ERSPC: = European Randomized Study of Screening for Prostate Cancer
PCP: = Primary care physician
